# Old-Growth *Platycladus orientalis* as a Resource for Reproductive Capacity and Genetic Diversity

**DOI:** 10.1371/journal.pone.0056489

**Published:** 2013-02-08

**Authors:** Lin Zhu, Anru Lou

**Affiliations:** 1 State Key Laboratory of Earth Surface Processes and Resource Ecology, College of Life Sciences of Beijing Normal University, Beijing, China; CNR, Italy

## Abstract

**Aims:**

*Platycladus orientalis* (Cupressaceae) is an old-growth tree species which distributed in the imperial parks and ancient temples in Beijing, China. We aim to (1) examine the genetic diversity and reproductive traits of old-growth and young populations of *P. orientalis* to ascertain whether the older populations contain a higher genetic diversity, more private alleles and a higher reproductive output compared with younger populations; (2) determine the relationships between the age of the population and the genetic diversity and reproductive traits; and (3) determine whether the imperial parks and ancient temples played an important role in maintaining the reproductive capacity and genetic diversity of *Platycladus orientalis.*

**Methods:**

Samples from seven young (younger than 100 yrs.) and nine old-growth (older than 300 yrs.) artificial populations were collected. For comparison, three young and two old-growth natural populations were also sampled. Nine microsatellite loci were used to analyze genetic diversity parameters. These parameters were calculated using FSTAT version 2.9.3 and GenAlex v 6.41.

**Important Findings:**

The old-growth artificial populations of *P. orientalis* have significantly higher genetic diversity than younger artificial populations and similar levels to those in extant natural populations. The imperial parks and ancient temples, which have protected these old-growth trees for centuries, have played an important role in maintaining the genetic diversity and reproductive capacity of this tree species.

## Introduction

Old-growth tree populations are assumed to be potential resources of genetic diversity and high reproductive output; however, there is little relevant research and empirical evidence for this assertion [1], [2], [3]. The research regarding old-growth trees has mostly concentrated on species diversity and distribution [4], [5], the relationship of the growth status and soil condition [6], [7], [8], cultivation, maintenance and rejuvenation [9], [10]. Studies of red spruce (*Picea rubens*) have shown positive relationships among the age, stem diameter, height and genetic and reproductive status of old-growth and older stands of *P. rubens*, and old-growth forests may serve as reservoirs of genetic diversity and seed sources to maintain populations under the pressures of climate change and population fragmentation [1]. The gene pool of aged tree populations holds a variety of valuable genes, such as those for resistance and other genes that may be very useful for sustaining the health of the species or artificial breeding in the face of environmental changes [11], [12].

A great number of imperial parks, tombs and ancient temples were established in Beijing, an old city with a long history as the capital of China. These sites provide a long-term stable environment (hundreds of years) for long-lived trees and protect the genetic diversity of these trees. Beijing City has the largest number of long-lived trees in the world. In a survey of ancient and famous trees in 2007, 28 families, 47 genera, 66 species and 39408 old-growth trees were found, 6122 of which were older than 300 years [5], including species of *Ginkgo biloba*, Chinese jujube (*Ziziphus jujuba*), *Sophora japonica*, *Pinus tabulaeformis*, and *Pinus bungeana*. The largest number of these trees (53.8%) consists of *Platycladus orientalis* (Cupressaceae), an evergreen tree species that originated in China, and that can live for centuries. Natural populations of *Platycladus orientalis* are rare and restricted to Beijing, Hebei, Shanxi and Shaanxi provinces [13]. However, due to its ability to thrive under a very wide range of climate and soil conditions, *P. orientalis* is an afforestation tree species which is widespread and naturalized throughout China and as an introduced species in both North Korea and eastern Russia [13]. Previous studies on *P. orientalis* have focused on its chemical components [14] and its drought-resistance characteristics [15]. Direct investigation of the genetic diversity and other characteristics of old-growth and young *P. orientalis* populations can help to determine the differences between these populations and provide empirical evidence for the assertion that the old-growth populations are genetically diverse and potential sources for obtaining seeds for conservation and reintroduction purposes.

Here we use *P. orientalis* to investigate the levels of genetic diversity and reproductive traits in old and younger artificial populations and compare these levels to those observed in five natural populations. We hypothesize that older populations will harbor higher genetic diversity than younger populations. Through this study, we will better understand the importance of protecting old-growth trees, both for their cultural significance as a biological archive [16] and for the gene pool that they represent. Furthermore, we will understand the role of these old populations in maintaining biodiversity. The objective of this study was to(1) compare the genetic diversity and reproductive traits (seed size and weight) of old-growth and younger populations of *P. orientalis* and to (2) determine the relationships between the age of the population and the genetic diversity and reproductive traits.

## Results

### Reproductive Capacity

Of the 241 individuals (52.28%) of the old-growth populations, 126 produced cones, whereas only 69 of the 187 individuals (36.90%) of the 100-yr. age group displayed cones. In the 300-yr. age group, the proportion of individuals with cones was significantly higher than the 100-yr. age group (Chi-square test; χ^2^  =  10.047; *p*  =  0.002). There was no significant relationship between the average value of the DBH of a population and the percentage of individuals with cones in the artificial populations.

**Figure 1:**
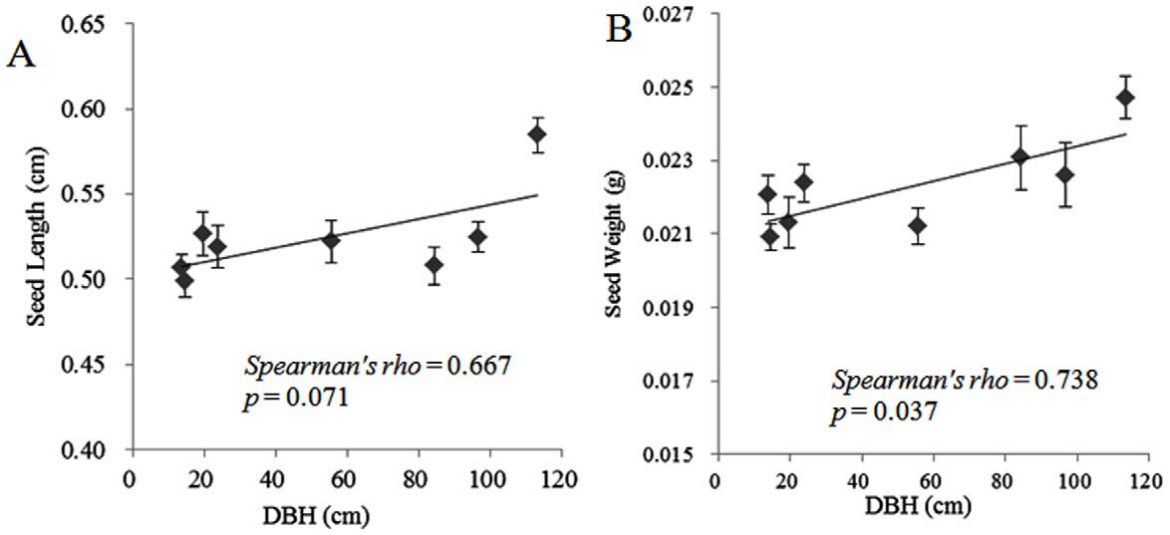
Relationships between the mean diameter at breast height (DBH) and (A) seed length and (B) seed weight of the eight sampled populations of *Platycladus orientalis*(mean ± S.E.).

There were no significant differences in the seed weight between the eight sampled populations (data not shown), except for the TMA population, which was significantly longer (0.5845 ± 0.0101 cm, mean ± S.E.) and heavier (0.0247 ± 0.0006 g, Mean ± S.E.) than the other seven populations (*p* < 0.05). Seed length (*p*  =  0.071; n  =  8; *Spearman’s rho*  =  0.667; marginally significant) and weight (*p*  =  0.037; n  =  8; *Spearman’s rho*  =  0.738) for these populations were positively correlated with the average values of the DBH (Fig. 1a, b): Older populations tended to produce heavier seeds.

### Genetic diversity parameters

The genetic diversity parameters of these sampled populations are shown in Table 1. The nine loci used were highly polymorphic. The old-growth population in Dingling Tomb exhibited the highest values for the parameters of the observed heterozygosity (Ho  =  0.805), expected heterozygosity (He  =  0.880), number of alleles (Na  =  15.111), number of effective alleles (Ne  =  9.586), Shannon’s information index (I  =  2.291) and number of private alleles (PA  =  9). The largest range of allele size was also detected in the 300-yr age group, the XSA population (allele range  =  55.889). Conversely, the lowest values for seven of the parameters were found in the artificial 100-yr. populations BDC (Ho  =  0.584), SGS (He  =  0.635, AR  =  4.213), DL (Na  =  7.222, I  =  1.599, PA  =  1) and DT (allele range  =  30.222); the lowest Ne was found in DJA (4.528).

**Table 1:** Genetic diversity parameters of the sampled populations of *Platycladus orientalis.*

Population	Ho	He	N_a_	N_e_	F	I	A_R_	No. ofPrivate Alleles	Allele range
GROUP 1									
HLS	0.638	0.758	8.222	4.712	0.173	1.685	4.325	6.000	35.889
JF	0.646	0.790	11.111	6.189	0.201	1.943	4.894	9.000	36.889
SGS	0.635	0.635	8.778	4.587	0.059	1.619	4.213	4.000	35.222
BDC	0.584	0.748	10.667	5.552	0.234	1.839	4.670	4.000	38.556
DL	0.526	0.723	7.222	4.651	0.274	1.599	4.461	1.000	30.556
DT	0.481	0.728	9.111	4.646	0.349	1.690	4.338	4.000	30.222
RT	0.597	0.718	10.222	4.722	0.193	1.716	4.346	3.000	39.444
Mean	0.587	0.729	9.333	5.008	0.212	1.727	4.464	4.429	35.254
SE	0.058	0.045	1.297	0.573	0.083	0.114	0.220	2.321	3.358
GROUP 2									
WFA	0.704	0.775	13.000	5.613	0.110	1.997	4.938	1.000	41.778
DLA	0.805	0.880	15.111	9.586	0.089	2.391	5.863	9.000	44.889
DTA	0.708	0.808	11.667	6.561	0.156	2.029	5.091	8.000	47.778
TMA	0.739	0.843	14.000	7.686	0.137	2.208	5.401	6.000	49.778
ZSA	0.789	0.846	14.444	7.816	0.083	2.232	5.457	7.000	46.556
DJA	0.682	0.763	9.889	4.528	0.111	1.760	4.394	5.000	49.333
RTA	0.767	0.786	9.667	6.096	0.025	1.906	5.170	2.000	43.444
XSA	0.791	0.815	10.444	6.398	0.032	2.002	5.287	5.000	55.889
TTA	0.739	0.791	10.667	5.681	0.074	1.944	5.000	4.000	47.556
Mean	0.747	0.812	12.099	6.663	0.091	2.052	5.178	5.222	47.445
SE	0.041	0.036	1.966	1.412	0.041	0.181	0.383	2.485	3.886
GROUP 3									
ZF1*	0.759	0.820	11.556	7.696	0.082	2.017	5.282	6.000	43.222
ZF2*	0.740	0.812	13.000	8.321	0.074	2.131	5.351	7.000	41.889
SF*	0.745	0.829	10.778	7.138	0.097	2.060	5.332	7.000	35.111
Mean	0.748	0.820	11.778	7.718	0.084	2.069	5.322	6.667	40.074
SE	0.008	0.007	0.921	0.483	0.010	0.047	0.029	0.471	3.551
GROUP 4									
SFA*	0.789	0.830	9.556	5.893	0.018	1.861	5.229	8.000	31.000
ZFA*	0.701	0.834	8.333	5.910	0.101	1.870	5.209	2.000	47.889
Mean	0.745	0.832	8.945	5.902	0.060	1.866	5.219	5.000	39.445
SE	0.044	0.002	0.611	0.009	0.042	0.005	0.010	3.000	8.444

* Natural populations.

Group 1: 100-yr. artificial populations (Group 1); Group 2: 300-yr. artificial populations; Group 3: 100-yr. natural populations; Group 4: 300-yr. natural populations. Ho  =  Observed heterozygosity; He  =  Expected heterozygosity; Na  =  the average number of alleles; Ne  =  effective number of alleles; F  =  inbreeding coefficient; I  =  Shannon’s information index; A_R_  =  allele richness.

There was no difference between the old-growth artificial tree populations (Group 2) and natural populations (Groups 3 and 4) for the Ho, He, Na, Ne, I and AR parameters (*p* > 0.1). The allele range of Group 2 was significantly higher than that of the other three groups (*p* < 0.05). The Ho, H_E_, N_A_, N_E_, I and A_R_ values for Group 1 were significantly lower than those of the other groups (*p* < 0.05), but no difference was detected in the allele range between Group 1 and natural Groups 3 and 4. The private alleles of these groups did not differ significantly.

**Figure 2:**
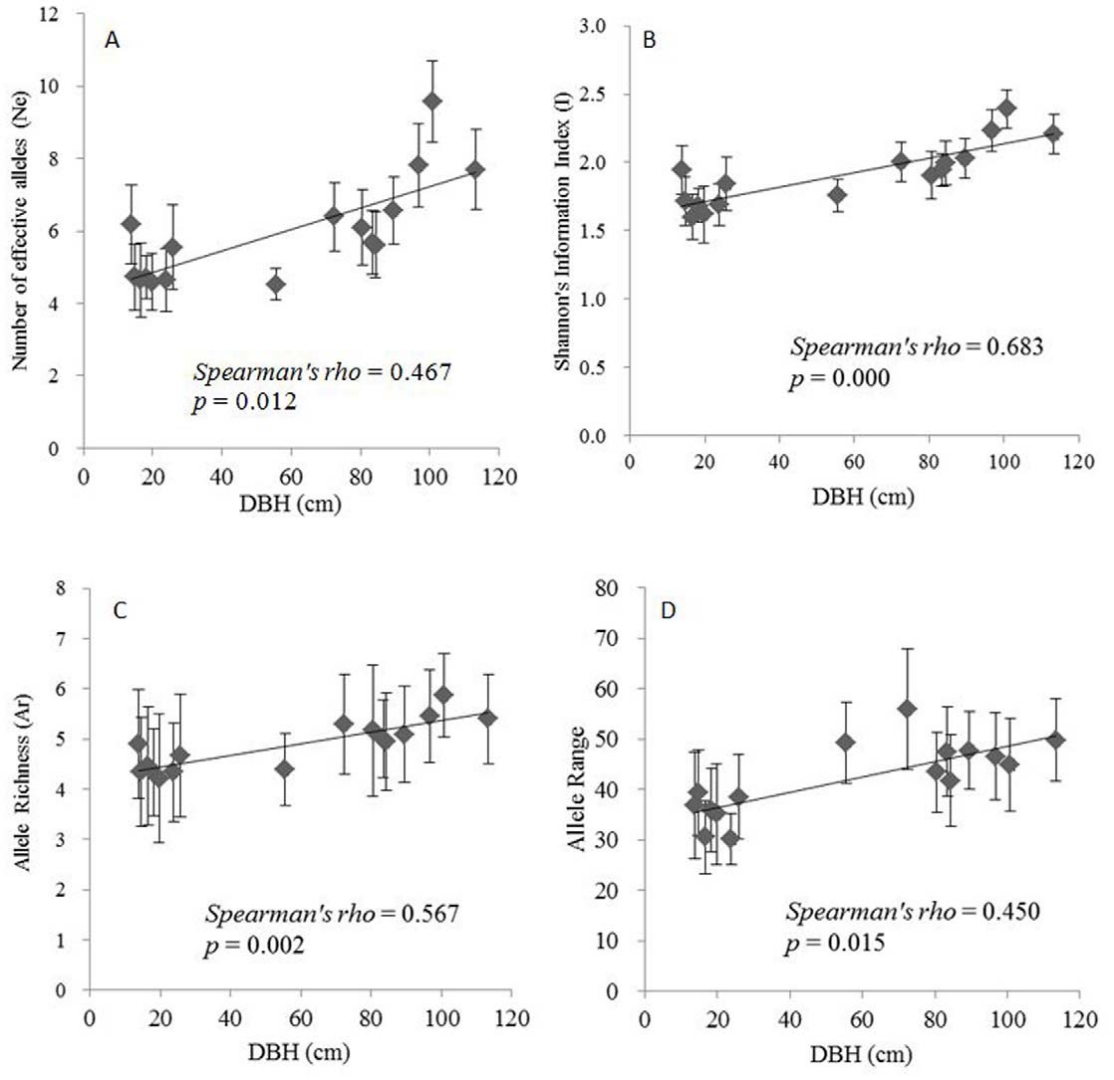
Relationship between the values of the mean diameter at breast height (DBH) and number of effective alleles (A), Shannon’s information index (B), allele richness (C) and allele range (D) in artificial populations of *Platycladus orientalis*(mean ± S.E.).

**Figure 3:**
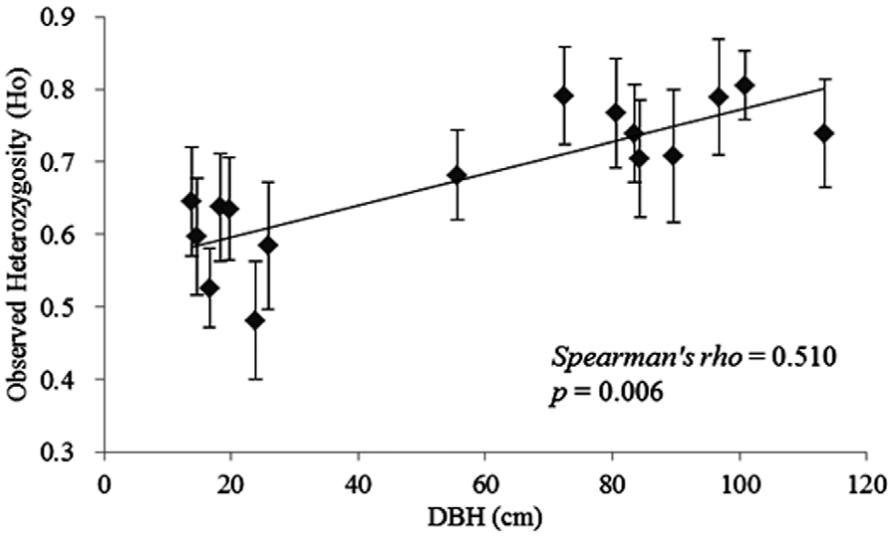
Relationship between the mean values of the diameter at breast height (DBH) and observed heterozygosity (Ho) in artificial populations of *Platycladus orientalis*(Mean ± S.E.).

The results of the DBH measurements indicate that values of the natural old-growth populations (Group 4) were not as large as the ancient artificial individuals of *P. orientalis*, probably because of environmental stress, such as drought and competition with other species. No relationship was found between the DBH and genetic diversity parameters. In the artificial populations, conversely, significant correlations were found between the population average values of the DBH and the average genetic diversity parameter values of the number of effective alleles (*Spearman’s rho*  =  0.467; n  =  16; *p*  =  0.012), Shannon’s information index (*Spearman’s rho*  =  0.683; n  =  16; *p*  =  0.000), allele richness (*Spearman’s rho*  =  0.567; n  =  16; *p*  =  0.002) and allele range (*Spearman’s rho*  =  0.450; n  =  16; *p*  =  0.015) (Fig. 2a, b, c and d). There was a strong positive relationship between the observed heterozygosity and population averages of the DBH (*Spearman’s rho*  =  0.510; n  =  16; *p*  =  0.006) (Fig. 3).

## Discussion

Old-growth tree populations can provide long-term stable environments for other species, such as animals and other plants, and forests can play an important role in the soil and water retention [17]. However, the role of these tree populations as genetic diversity reservoirs and seed sources has not been well considered and investigated [1]. In this study, we focused on the comparison of old-growth and young populations of *P. orientalis*. According to our results for seed trait measurements and microsatellite experiments, the 300-yr. artificial age group exhibited the highest reproductive traits and can represent most of the genetic diversity of this species. In addition, the 300-yr. artificial age group and the natural groups showed much higher levels of genetic diversity than the artificial 100-yr. age group.

### Relationship between the population DBH and Reproductive Capacity

Factors affecting reproductive success include fruit yield, seed weight and size, and seed quality directly determining the perpetuation of seed plant species [18]. Our examination of the cone set revealed that the natural populations did not reproduce as well as the artificial populations, probably because of the lack of water and the competition from other species. The sampled natural populations on the Zhuifeng and Shangfang mountains coexisted with Chinese pine (*Pinus tabulaeformis*) and *Robinia pseudoacacia* forests and some forest shrub, with some of the *P. orientalis* trees even growing on rocks. Therefore, their growth status, as based on the diameter at breast height and crown, and reproductive output, as based on the cone set, were lower than the artificial trees in the parks and other urban locations, which have adequate space, water and nutrients and were very well protected. Although the fruit-setting percentage of tree populations may have a positive relationship with the DBH [19], in the present study, there was no obvious correlation between the proportion of individuals with cones and the DBH of a population. However, the cone set was significantly higher in the 300-yr. than the 100-yr. artificial population, indicating that the reproductive capacity of the Group 2 was greater than Group 1.

Seed weight and size have a significant impact on seed germination, whereby the larger or heavier the seed is, the earlier and faster it germinates, which can be associated with higher reproductive fitness [18], [20], [21]. In our study, seed length and weight were positively correlated with DBH; in other words, the seeds sampled from the old-growth artificial populations were longer and heavier. Therefore, the 300-yr. age artificial group in the parks and tombs exhibited higher reproductive success. In future studies, seed germination experiments should be conducted to examine seedling traits to understand better the reproductive characteristics of different age groups of *P. orientalis*.

### Relationship between population DBH and Genetic diversity parameters

In long-living forests, the genetic diversity parameters could be significantly correlated with the tree growth traits, such as the DBH and height [1]. In our study, five of the genetic diversity parameters (N_E_, A_R_, I, allele range and Ho; Figs. 2 and 3) were positively correlated with the DBH for the artificial old-growth populations (Group 2) in the parks and temples, sites where the living conditions were probably more benign than in natural populations. These results supported the relationship between the *P. orientalis* population growth and genetic diversity. The higher genetic diversity in the artificial 300-yr. age group showed that the resources of individuals in these populations may be quite extensive [22].

Almost all of the mean values for the genetic diversity parameters of the old-growth artificial populations were similar to those in the natural populations (except for the allele range, which was remarkably larger than in the natural populations), indicating that these ancient artificial populations contain similar levels of the genetic diversity of natural populations of *P. orientalis*. In contrast, the genetic diversity parameters for the artificial young populations were significantly lower than other populations, indicating that, although the number of artificial young *P. orientalis* was the largest, in terms of the genetic diversity, this group was much less important than the old-growth and natural populations.

None of the genetic estimates obtained for the natural 100-yr. and 300-yr. age groups were notably different, and there were no relationships found between these estimates and the population average height or DBH, which may have resulted from the small number of sampled natural individuals. In Beijing, the amount of natural populations were limited, despite the large numbers of individuals of *P. orientalis* [5]. Therefore, the artificial populations play an important role in sustaining the species.

### Importance of parks and temples in the protection of old-growth trees and their reproductive capacity and genetic diversity

As the number of people living in large cities increases with the modern-day urbanization process, the restoration, protection and enhancement of the biodiversity in urban areas become more important [23]. The biodiversity in urban areas has a positive impact on the residents’ education; some research has shown that people who are exposed to environments with natural elements, such as flowers, trees and birds, will pay more attention to environmental issues [24]. The urban ecosystem is of great significance in the global genetic diversity and structure. For instance, rare plant or animal species can be raised in cities, thus protecting the genetic information of these species [23]. In such a large city with a long history as Beijing, many ancient temples, tombs, royal gardens and parks, and old tree populations and other in situ conservation sites of plant resources can enlarge the effective population size and enhance the gene flow between isolated populations [25]. If the plant populations within these city areas contain genetic variations that have been long lost in natural populations, then the preservation of artificial populations will be particularly necessary [25]. In our study, the ancient artificial populations contained a number of private alleles; in particular, population DLA exhibited nine private alleles, more than any natural population. Moreover, the estimates of the allele range in the artificial old-growth populations were the largest across all of the populations. From these results, we can infer that the long-living artificial populations may hold valuable genes that do not exist in nature.

The potential negative influences of artificially cultivated plants on the gene pool of the species also require attention: the genetic contamination of natural populations may produce negative effects on the fitness of the natural plants as a result of outbreeding depression [26], [27], [28]. Nonetheless, research regarding this aspect showed that this kind of contamination was rare or nonexistent [25].

None of the genetic diversity parameters obtained from the old-growth artificial populations sampled in our study differed from those of the natural populations, and some of them showed an even higher level of genetic diversity than the populations in nature. Moreover, the artificial 300-yr. age group had a greater reproductive capacity than the young artificial and natural populations. These results suggest that the old-growth *P. orientalis* populations in the urban areas can serve as reservoirs for genetic diversity and seed sources for this species [29]. These populations are of great importance and necessity, and maintain a high level of genetic diversity for the species, possibly serving as a buffer against the loss of genetic variation in the natural populations [25].

As garden plants, the artificial populations maintained alleles similar to the nearby natural populations and may hold a higher level of genetic diversity [25]. In population DLA, Ho, Ne, allele richness and I values were the highest in the 300-yr. population. These results may be explained by the seed resources: these trees were planted hundreds, even a thousand years ago, and their seeds may have been recruited from the soil-stored seed bank that was established prior to urbanization or from the populations far from the city [30].

These royal gardens, imperial tombs and ancient temples are in a solemn atmosphere and thus they protected these old trees which have been given extraordinary significance from deforestation, and indirectly protected their valuable genes. These urban areas play an important role in maintaining seed availability and genetic diversity of *Platycladus orientalis*.

## Materials and Methods

### *Platycladus orientalis* Populations and Seeds Sampling

According to the records from the parks and temples [31], we studied seven young (younger than 100 yrs.) and nine old-growth (older than 300 yrs.; tagged by the Beijing Municipal Bureau of Landscape and Forestry) artificial populations. The living-conditions of these artificial populations were similar in population density, temperature and light intensity. For comparison, three young and two old-growth natural populations in the Beijing area were sampled in 2011 (Table 2); the small number of sampled natural populations was because the natural populations were rare in Beijing. The cone set (the proportion of reproductive individuals in a population) and diameters at breast height (DBH) of individuals were recorded. The seed lengths and weights of four young artificial populations and four old-growth artificial populations, which were randomly selected (Table 3), were measured using the sampling strategy of randomly selecting approximately 10 individuals in a population and collecting five cones from each chosen individual. There were four groups of populations: 100-yr. artificial populations (Group 1), 300-yr. artificial populations (Group 2), 100-yr. natural populations (Group 3) and 300-yr. natural populations (Group 4).

**Table 2:** Sampling sites, age groups and number of individuals of *Platycladus orientalis* populations in Beijing.

Age Group	Site	Abbreviation	Number of Samples	Latitude and Longitude
300 yr	Zhongshan Park	ZSA	30	39°54’40.6”N, 116°23’41.0”E
	The Altar to the Sun	RTA	11	39°54’52.8”N, 116°26’12.1”E
	Temple of Earth	DTA	31	39°57’22.6”N, 116°24’55.9”E
	Dajue Temple	DJA	29	40°03’17.2”N, 116°07’01.0”E
	Wofo Temple	WFA	31	40°00’06.7”N, 116°12’29.6”E
	Xiangshan Park	XSA	16	39°59’09.2”N, 116°11’35.1”E
	The Ancestral Temple	TMA	39	39°54’41.8”N, 116°23’59.8”E
	Dingling Tomb	DLA	24	40°17’49.8”N, 116°13’20.8”E
	Temple of Heaven	TTA	30	39°53’01.3”N, 116°24’46.2”E
	Mt. Zhuifeng	ZFA*	15	40°25’57.1”N, 117°08’27.2”E
	Mt. Shangfang	SFA*	10	39°40’40.0”N, 115°49’43.7”E
100 yr	The Altar to the Sun	RT	25	39°54’51.3”N, 116°26’40.2”E
	Temple of Earth	DT	30	39°57’08.1”N, 116°24’47.6”E
	Dingling Tomb	DL	24	40°17’39.7”N, 116°13’32.2”E
	Badachu	BDC	30	39°56’33.7”N, 116°11’24.5”E
	Mt. Jiufeng	JF	30	40°03’39.2”N, 116°05’44.7”E
	Mt. Hongluo	HLS	25	40°23’41.9”N, 116°37’18.5”E
	Mt. Shugu	SGS	22	40°18’56.3”N, 116°48’56.5”E
	Mt. Zhuifeng 1	ZF1*	20	40°25’37.0”N, 117°08’16.9”E
	Mt. Zhuifeng 2	ZF2*	23	40°26’58.0”N, 117°07’29.5”E
	Mt. Shangfang	SF*	20	39°40’35.7”N, 115°49’42.1”E

* Natural populations.

Population name ending with an “A” means the population was old-growth (300-yr.); otherwise the population was young (100-yr.).

**Table 3:** Number and location of the trees used for cone collection in the artificial populations of *Platycladus orientalis*.

	300 yrs.				100 yrs.			
	WFA	TMA	ZSA	DJA	DT	RT	SGS	JF
Number of individuals	9	10	8	8	9	7	8	9
Total	35				33			
Number of cones	47	51	45	44	45	34	39	41
Total	187				159			

### Genetic Diversity Analysis

Total genomic DNA was extracted from silica gel–dried leaves from individuals’ specimen of *P. orientalis* with a Plant Genomic DNA Kit (Tiangen Biotech, Beijing, China). Nine microsatellite loci and the same PCR condition as in reference [32] were used to analyze the genetic diversity parameters, including the average number of alleles (Na), effective number of alleles (Ne), Shannon’s information index (I), observed heterozygosity (Ho), expected heterozygosity (He), allele richness (A_R_) and allele range. These parameters were calculated using FSTAT version 2.9.3 [33]and GenAlex v 6.41 [34].

### Statistical Analysis

The average numbers of the population trait - DBH were calculated. To determine whether older populations harbor higher genetic diversity, we estimated the correlation between DBH, a proxy of age in this species, and genetic diversity parameters using bivariate correlations in SPSS version 16.0. The relationships among the population average DBH and seed size were also analyzed by bivariate correlations. The differences in genetic diversity parameters between the age groups were analyzed using a one-way ANOVA, followed by post-hoc pairwise comparisons between groups using a Bonferroni correction. The differences among groups for reproductive capacity were examined using the Chi-square test.
